# Healthcare providers’ views on the delivery of preconception care in a local community setting in the Netherlands

**DOI:** 10.1186/s12913-017-2051-4

**Published:** 2017-01-31

**Authors:** M. Poels, M.P.H. Koster, A. Franx, H.F. van Stel

**Affiliations:** 1 Division Woman and Baby, University Medical Center Utrecht, P.O. Box 85090, Utrecht, 3508 AB The Netherlands; 2 Department of Health Technology Assessment, Julius Center for Health Sciences and Primary Care, University Medical Center Utrecht, P.O. Box 85500, Utrecht, 3508 GA The Netherlands; 3 Present Address: Department of Obstetrics and Gynecology, Erasmus University Medical Center, P.O. Box 2040, Rotterdam, 3000 CA The Netherlands

**Keywords:** Preconception care, Qualitative research, Healthcare providers, Healthcare delivery, Implementation strategy

## Abstract

**Background:**

The attention for preconception care (PCC) has grown substantially in recent years, yet PCC is far from routine in daily practice. One of the major challenges for the implementation of PCC is to identify how it can best be organized and provided within the primary care setting. The aim of this study was to identify bottlenecks and solutions for the delivery of PCC from a healthcare providers’ perspective in a local community setting in the Netherlands.

**Methods:**

Health professionals within the region of Zeist, the Netherlands, were invited for a meeting on the local implementation of PCC. Five parallel group sessions were held with 30 participants from different disciplines. The sessions were moderated based on the Nominal Group Technique, in which bottlenecks (step 1) and solutions (step 2) for the delivery of PCC were gathered, categorized and prioritized by the participants.

**Results:**

Participants expressed that the provision of PCC is challenging due to lack of awareness, the absence of a costing structure and unclear allocation of responsibilities. The most pragmatic approach considered was to make interdisciplinary arrangements within the local primary care setting. Participants recommended to 1) settle a costing structure by means of third party reimbursement, 2) improve collaboration by means of a local cooperation network and an adequate referral system, 3) invest in education, tools and logistics and 4) increase uptake rates by the routine opportunistic offer of PCC and promotional campaigns.

**Conclusions:**

From a provider’s perspective a tailored approach is advocated in which interdisciplinary arrangements for collaboration and referral are set up within the local primary care setting.

## Background

Preconception Care (PCC) is targeted at couples contemplating pregnancy and aims to enhance the future health of mother and child. PCC focuses on the period prior to conception and is therefore not embedded in routine antenatal care. The purpose of PCC is to identify and reduce biomedical, behavioral and social risk factors that are present during the preconception phase, such as smoking, alcohol consumption, medication use and hereditary diseases [1]. PCC has three principal components: risk assessment, health promotion and interventions. Examples of such interventions are folic acid supplementation, treatment of infections and smoking cessation programs [2–4]. PCC can be delivered in multiple ways. PCC can be aimed at individual parents-to-be, such as tailored consultations or collectively targeted at all women of childbearing age through community-based public health programs, such as national folic acid campaigns [2, 5, 6]. Shannon et al. performed a systematic review on the delivery of PCC and distinguished four main settings in which PCC can be delivered: primary care, hospital-based, preconception care clinics and high-risk care/community outreach. Of those, individual consultations during general practice visits were identified as the most frequent strategy of PCC [6].

The delivery of PCC depends on the context of national and local health systems, as services, facilities, financial and organizational structures may influence the way PCC is offered [7]. In many European countries, PCC is still an emerging concept, while there is a national agenda for preconception health and health care in the United States [8]. In most countries PCC has not become part of routine practice and the uptake remains low (rates varying between 27 and 39%) [9–12]. The heterogeneity of health care systems calls for regional approaches to enhance the delivery of PCC [6–8].

Previous research suggests that factors hindering the delivery of PCC contribute to the general low uptake of PCC. In a Dutch study by Heyes et al. it was found that these inhibiting factors include resource constraints, lack of training and practice policies and procedures, and difficulty in targeting couples planning conception [13]. While studies from several Western countries found positive attitudes towards delivering PCC among general practitioners (GPs), midwives and obstetrician-gynecologists, the incorporation of PCC in daily practice is challenging [12, 14–18]. One of the major difficulties is that prospective parents are hard to reach by healthcare providers who offer PCC [4, 19]. In the Netherlands, GPs have the most frequent contact with reproductive aged women, yet are less engaged in pregnancy related issues since midwives conduct primary pregnancy care. Moreover, GPs think of PCC as a time consuming form of care with limited proof of its effectiveness and necessity [20]. Meanwhile, previous research shows that the majority of midwives feels responsible to provide PCC, yet the antenatal booking visit generally takes place at the end of the first trimester of pregnancy, which makes it difficult for them to reach women with childbearing plans [17, 18]. A recent Dutch study by M’Hamdi et al. emphasized the need for further research on those organizational barriers and how interdisciplinary collaboration and referral can lead to tailored intervention approaches [20]. Therefore, the objective of this study was to identify bottlenecks and solutions for the delivery of PCC from a healthcare providers’ perspective in a local community setting in the Netherlands.

## Methods

### Setting

The study took place in Zeist, a middle-sized municipality with approximately 60,000 inhabitants, which is located in the center of the Netherlands [21]. In Zeist, 77% of the population has a Dutch ethnicity whereas 13% has a non-western ethnicity, which is representative for the Netherlands. Educational and income levels in Zeist are high compared to national figures [22, 23]. Zeist has a birth rate of 10.5 per 1000 inhabitants and one primary care midwifery center [21].

The Dutch healthcare system has three levels of care: primary, secondary and tertiary care [24]. Women who are indicated for low risk pregnancies and deliveries receive care at the primary care level by independently practicing midwives [25]. In 2007, the Dutch Health Council recommended to integrate PCC in the health care system and subsequently, guidelines for GPs and midwives and risk assessment instruments were developed [26, 27]. Zwangerwijzer, a validated self-administered pre-pregnancy internet-based questionnaire, is publicly available and free-of-charge [27]. Prospective parents can send the results of this checklist to a healthcare provider to verify risks and provide preconception advice [28]. Yet, collective reimbursement of PCC in Dutch obstetric care has not been arranged up to now. There is no coverage for PCC consults within the mandatory basic healthcare insurance and although GPs can get reimbursed for time spent on PCC advice, midwives have no means for financial compensation. Moreover, the existing registration systems have limited possibilities to register on PCC and there is no standard reporting format or electronic patient file which allows referral between GPs and midwives.

### Participants

In June 2014, a meeting on how to locally provide PCC took place in the municipality Zeist, the Netherlands. Health professionals (*n* = 146) with a relation to maternal and/or child health within the region of Zeist received a written invitation to participate in the meeting, either directly or via key contacts within the regional healthcare system. We recruited participants from the following disciplines: midwifery, obstetrics and gynecology, fertility, general practice, preventive child health care, maternity care, physiotherapy, pharmaceutics, dietetics and policy makers. We used a convenience sample of professionals who were willing to participate and strived to reach a good representation of all disciplines by sending reminders or directed personal invitations. The participant group consisted of 30 healthcare providers, which were diversely distributed among the discussion groups, according to their professional background (Table 1). The participation of pharmacists and general practitioners was limited, which was reported to be caused by busy schedules and limited interest in PCC. The study has been approved by the Medical Ethical Review Board of the UMC Utrecht (protocol no. 13–475) and all participants gave informed consent to participate.

**Table 1:** Distribution of health disciplines among discussion groups

	Invited	Participated in discussion group					Total
		A	B	C	D	E	30
General practitioners	36			1		1	2
Midwives	31	2	2	2	1	2	9
Gynecologists	18	1				1	2
Physiotherapists	17	1	1		1	1	4
Fertility specialists	10	1	1	1	1		4
Pharmacists	10						0
Preventive child healthcare	7	1		1	1		3
Maternity care	7		1			1	2
Dieticians	5			1			1
Municipal policy officers	3		1				1
Patient advocacy	2				2		2

### Data collection

The meeting contained an educational part, followed by group discussion sessions. Five parallel discussion sessions were held, each guided by an experienced moderator. To minimize inter-group variety, a preparation session was held and a detailed protocol was used to guide the sessions (Table 2). The aim of the sessions was to identify bottlenecks (part one) and solutions (part two) for the regional delivery of PCC.

**Table 2:** Protocol discussion groups

Time	Phase	Activity
Part 1: Bottlenecks		
5 min	Introduction	The moderator introduces him/herself and invites the participants to introduce themselves shortly by providing their name, current job position and employer.
5 min	Introduction	The moderator gives a short explanation about the procedures during the discussion group: - Role of the moderator - Conversation rules - Use of flipchart and post-its - Structure: part 1 bottlenecks, part 2 solutions
5 min	Generating ideas	The moderator poses the central question: *“What are your experiences with PCC in the local situation? What bottlenecks do you perceive or experience?”* Participants get 3–4 min to write down their ideas in silence on post-its.
10 min	Recording ideas	The moderator invites the participants one-by-one to share their ideas. There is no room for discussion yet, first all ideas will be collected. The post-its are randomly put on the flipchart.
15 min	Discussing ideas	The moderator opens the discussion by asking the participants to respond and elaborate on the collected ideas. The moderator guides the discussion by asking questions. Participants are requested to write down new/additional ideas on post-its, which are added to the flipchart.
10 min	Discussing ideas	The moderator asks the participants to identify common themes among the ideas and write these down on the flipchart, together with some catchwords. The post-its are clustered accordingly.
5 min	Voting on ideas	The moderator requests the participants to individually prioritize the bottlenecks. The themes are listed by number on a separate flipchart. Each participant makes a ranking by allocating a relative score to each theme, in which 1 is the highest priority, 2 is the second highest priority, and so on.
5 min	Voting on ideas	The moderator collects the individual rankings and combines the scores on a sheet to establish a collective ranking. The result is shared with the participants.
10 min		Break During the break the moderators meet and share which themes received most emphasis in their group during the first part. Accordingly, the six predefined themes are distributed among the groups for discussion during the second part. Themes: 1. finances and time 2. providing and organizing PCC 3. collaboration between healthcare providers 4. logistics and tools 5. knowledge and education of healthcare providers 6. reaching the target population.
Part 2: Solutions		
5 min	Generating ideas	The moderator explains that the second part of the discussion group will emphasize on 1–2 themes which are distributed among the groups to elaborate on specific solutions. The moderator explains the theme (s) and poses the question: *“What are your ideas to improve the delivery of PCC in the local situation?”.* Participants get 3–4 min to write down their ideas in silence on post-its.
10 min	Recording ideas	The moderator invites the participants one-by-one to share their ideas. There is no room for discussion yet, first all ideas will be collected. The post-its are randomly put on the flipchart.
15 min	Discussing ideas	The moderator opens the discussion by asking the participants to respond and elaborate on the collected ideas. The moderator guides the discussion by asking questions. Participants are requested to write down new/additional ideas on post-its, which are added to the flipchart.
10 min	Discussing ideas	The moderator asks the participants to identify common themes among the ideas and write these down on the flipchart, together with some catchwords. The post-its are clustered accordingly.
5 min	Wrap-up	The moderator wraps up the discussion by giving a short summary of the discussion and providing highlights.

The Nominal Group Technique (NGT) was used to guide this study. NGT is a method to gather information and gain consensus through small-group discussion sessions. The principle of NGT allows all group members to participate equally and contribute their ideas and suggestions to the discussion. The steps used during NGT prevent the domination of a single person during the sessions. Moreover, it limits possible researcher bias, as data interpretation is for the larger part performed by participants. NGT generally entails four subsequent phases: “generating ideas”, “recording ideas”, “discussing ideas” and “voting on ideas”. During the first phase, the moderator presents a central question and participants are requested to write down their ideas independently in silence. Next, a feedback session is held in which participants are encouraged to share their ideas, yet without discussing them. Each new idea is recorded on a flip chart by the moderator until all ideas are collected. During the third phase, group discussion takes place to clarify and elaborate on gathered ideas and to determine their relative importance. In the final phase each participant prioritizes the ideas by independently allocating scores. The moderator combines those scores and establishes a collective ranking [29].

The sessions were structured around six pre-defined themes, which we derived from the literature: 1) finances and time; 2) providing and organizing PCC; 3) collaboration between healthcare providers; 4) logistics and tools; 5) knowledge and education of healthcare providers and 6) reaching the target population [30]. The discussion sessions took 90 min, consisting of two parts of 45 min with a short break of 15 min in between. The sessions had an emphasis on soliciting individual input, followed by group discussions. Part one (identifying bottlenecks) was the same in all five groups. In part two (solutions), each group discussed a different topic, which was decided upon by the moderators during the break, based on the bottlenecks identified in the first part of the session.

### Data analysis

The method of NGT allows participants to contribute to the analytic process, as participants cluster and prioritize ideas. Thematic analysis was used to further analyze the data retrieved during the group discussion sessions. This method provides to identify, analyze and report patterns within data, while retaining a detailed data set [31]. All ideas that were collected on the flip charts and post-its during each discussion session were extracted verbatim as raw data into a single database file. We distinguished three levels of abstraction during analysis: (1) data (i.e. the collected ideas), (2) topics (clusters of raw data based on their content) and (3) themes (clusters arranged by the six pre-defined themes). Initially, one author (MP) reviewed all data to identify topics and set up a coding scheme to allocate topics and corresponding themes. There was also a possibility to add new themes, yet all emerging topics fitted one of the existing themes. Initial analysis was performed by one author (MP), while extraction and coding was performed by the first author and verified by a second author (HvS). Discrepancies were discussed until consensus was attained and both authors agreed on final analyses. Each discussion group used their own topics to complete the ranking assignment. To allow for comparison between groups, we categorized the ranked topics into themes using the coding scheme. In some groups, multiple topics were administered to one theme. Therefore, total weighted scores were calculated, based on the number of topics and participants. Accordingly, a top priority was established for each group and combined to a collective ranking score with a top six priority of bottlenecks for the delivery of PCC.

## Results

### Finances and time

Participants indicated the absence of a costing structure to be an important barrier for the provision of PCC. PCC consults are expected to be time consuming, which limits the feasibility of incorporating it into regular consults. Given that reimbursement options are currently absent, providing a PCC consult without compensation was not considered lucrative. On the other hand, invoicing PCC consults to prospective parents was expected to have an adverse effect on the already low uptake rates. Third party reimbursement was the main solution provided by participants to settle financial difficulties. Suggested measures included contracts with individual healthcare insurers, arranging full compensation within basic health insurance and obtaining municipal support to raise funds for local implementation of PCC.

### Providing and organizing PCC

In all discussion groups, participants felt it was unclear who should be the entitled provider for PCC. General practice was regarded the most suitable setting to address preconception health issues, as GPs most frequently encounter women of childbearing age. Yet, GPs were underrepresented at the meeting. Participants expressed that GPs are not as interested in PCC issues due to the organization of the local healthcare system in which GPs are pressured by their gatekeeper function and midwives conduct primary pregnancy care. The most pragmatic approach considered was to make local arrangements in which the most involved party is appointed to conduct PCC consults, preferably within a primary care setting. In the local setting of Zeist, midwives were considered to have less access to women with childbearing plans compared to GPs, yet are the most willing and therefore most eligible party to provide PCC. Suggestions were made to offer PCC both individually and collectively. Individual consults were advocated to be offered at a fixed time and location by one or two midwives (or other appointed providers) who are trained in PCC. Additionally, broader informational sessions at high schools, healthcare or municipal facilities could serve to address a wider public.

### Collaboration between healthcare providers

Participants experienced the level of collaboration between healthcare providers with regard to PCC to be limited, at least in the local setting of the municipality Zeist. This was attributed to both a lack of awareness of PCC and existing tensions between different healthcare disciplines. In the local setting, it is generally unknown which collaboration partners can be addressed for general or specific preconception health advice. Since referrals for preconception advice to GP’s or other providers were considered rare, this is a self-sustaining bottleneck. In order to facilitate collaboration between healthcare providers, it was suggested to set up a clear network of collaboration partners for preconception health issues and to design care pathways. Responsibilities could be shared, as all involved healthcare providers can be assigned the role of either referrer or provider of PCC.

### Logistics and tools

Succeeding the discussion of the previous themes during the sessions, it became evident that a comprehendible overview of relevant medical conditions, collaborators and tools/guidelines for PCC is lacking. Although the “Zwangerwijzer” tool can be very useful and timesaving in preparing preconception care consults, familiarity among healthcare providers is limited. Moreover, IT-solutions to register, refer and exchange client details for PCC were considered necessary. During the group discussion sessions it was suggested to compose a clear protocol for PCC, containing indications, referral possibilities and collaboration agreements. A social map that provides an overview of all (local) collaboration partners was considered useful.

### Knowledge and education of healthcare providers

Participants indicated that healthcare providers fall short in their knowledge of PCC. Although some of the participating midwives were educated on PCC, it was recognized that formal professional education on PCC in the Netherlands falls short. Moreover, the aforementioned lack of awareness was considered an important factor, as well as not being convinced of the importance, need, benefits and efficacy of PCC. Participants expressed that many healthcare providers restrict themselves to their own discipline and are less likely to invest in general health promotion, such as PCC. Since there is not much routine in conducting PCC consults or giving preconception advice, participants felt there are few opportunities to build expertise. Healthcare providers were considered not enough experienced and equipped to provide preconception advice. Investing in education and organizing refresher courses were proposed to improve healthcare providers’ knowledge and awareness of PCC. Equally important, it was encouraged to embed communication and interviewing skills in educational courses, as some healthcare providers experienced timidity and reluctance to discuss the highly personal issue of a (future) wish to conceive.

### Reaching the target population

In all discussion groups, participants pointed out the difficulty of reaching women of childbearing age, since the wish to conceive generally stays unspoken until women present when pregnancy already occurred. Participants felt that women’s unawareness and rather poor understanding of personal risks are the main contributors to low uptake rates. The common notion was that women who attend PCC are often those who have less need for preconception advice, since they are more engaged and already prepared. It was considered far more challenging to reach high-risk groups, especially women with low socioeconomic status, non-western ethnicity or living in deprived areas. Ignorance, lack of self-knowledge and inadmissibility for preconception information were considered key barriers for reaching the target population. Consequently, it was recommended to shift the provision of PCC from demand-driven to supply-driven, in order for PCC to be offered opportunistically by routinely discussing the subject during every suitable health encounter with both male and female clients of reproductive age. GPs, practice nurses, physiotherapists, pharmacists, dietetics and social workers all have the opportunity to casually address PCC. During those health encounters, the existence and relevance of PCC may be addressed, as the context of most treatment indications asks for further preconception advice. Moreover, it was encouraged to integrate PCC as a fixed protocol item during maternity care, the postpartum check-up at the midwifery practice, check-ups at the preventive child healthcare service and during contraceptive advice at the GPs office. Eventually, signaling and alerting couples for preconception advice could in this way become routine practice for a broad range of healthcare providers. Additionally, it was suggested to launch a promotional campaign to reach the target group. Examples were given to distribute marketing materials in public places, advertise in newspapers, magazines and TV-commercials, gain publicity, join local and community activities and use social media. Another suggestion was to recruit volunteers and key figures for PCC campaigns to raise awareness trough outreaching activities in communities and districts.

### Priority of bottlenecks

Table 3 shows the group ranking of bottlenecks for the delivery of PCC for each discussion group. Table 4 shows that collectively, financial & time constraints were acknowledged to be the most important, followed by the organization of PCC. Collaboration between healthcare providers and logistics & tools were regarded the least important.

**Table 3:** Group ranking of bottlenecks for the delivery of PCC for each discussion group

Group A	Participants						Total score	Weighted score	Priority
Themes	A1	A2	A3	A4	A5	A6			
Finances & time	2	3	1	1	1	1	9	1,5	1
Knowledge & education of healthcare providers	1	2	2	3	3	2	13	2,2	2
Reaching the target population	3	1	3	2	2	3	14	2,3	3
Collaboration between healthcare providers	4	4	4	4	4	4	24	4,0	4
Group B	Participants						Total score	Weighted score	Priority
Themes	B1	B2	B3	B4	B5	B6			
Finances & time^a^	13	10	3	11	14	7	58	4,8	1
Collaboration between healthcare providers^a^	10	9	14	10,5	3	15	61,5	5,1	2
Providing and organizing PCC^a^	12	11	11	14,5	14	11	73,5	6,1	3
Reaching the target population^a^	10	15	17	9	14	12	77	6,4	4
Knowledge & education of healthcare providers	9	9	8	8,5	7	9	50,5	8,4	5
Group C	Participants						Total score	Weighted score	Priority
Themes	C1	C2	C3	C4	C5	C6			
Reaching the target population	2	1	2	2	5	2	14	2,3	1
Providing and organizing PCC	1	4	1	1	3	4	14	2,3	1
Knowledge & education of healthcare providers	3	2	4	3	1	1	14	2,3	1
Finances & time	4	5	3	4	2	5	23	3,8	2
Logistics & tools	5	3	5	5	4	3	25	4,2	3
Group D	Participants						Total score	Weighted score	Priority
Themes	D1	D2	D3	D4	D5	D6			
Finances & time	3	1	1	6	1	1	13	2,2	1
Providing and organizing PCC	2	3	3	5	5	2	20	3,3	2
Reaching the target population^b^	10	11	12	6	9	12	60	3,3	2
Knowledge & education of healthcare providers^a^	7	8	9	5	9	10	48	4,0	3
Logistics & tools	7	7	5	7	7	7	40	6,7	4
Group E	Participants						Total score	Weighted score	Priority
Themes	E1	E2	E3	E4	E5	E6			
Knowledge & education of healthcare providers^a^	4	7	12	9	9	6	47	3,9	1
Finances & time	8	2	6	5	1	3	25	4,2	2
Reaching the target population^b^	14	12	7	11	21	14	79	4,4	3
Collaboration between healthcare providers	4	8	8	4	2	6	32	5,3	4
Logistics & tools	6	7	3	7	3	7	33	5,5	5

^a^Two items were scored for this theme, contributing to a higher total score

^b^Three items were scored for this theme, contributing to a higher total score

**Table 4:** Collective ranking of bottlenecks for the delivery of PCC

	Discussion group					Total score	Weighted score	Priority
Themes	A	B	C	D	E			
Finances & time	1	1	4	1	2	9	1,8	1
Providing and organizing PCC		3	1	2		6	2,0	2
Knowledge & education of healthcare providers	2	5	1	3	1	12	2,4	3
Reaching the target population	3	4	1	2	3	13	2,6	4
Collaboration between healthcare providers	4	2			4	10	3,3	5
Logistics & tools			5	4	5	14	4,7	6

## Discussion

This study set out to provide a providers’ perspective on bottlenecks and solutions for the delivery of preconception care in a local community setting in the Netherlands. Participants expressed that the provision of PCC is challenging due to lack of awareness, the absence of a costing structure and unclear allocation of responsibilities. The most pragmatic approach considered was to make interdisciplinary arrangements for collaboration and referral within the local primary care setting, A tailored approach was advocated, in which the responsibility for PCC is shared among a few appointed professionals who provide PCC, while all other relevant professionals act as referrers for PCC. In this way, multiple disciplines – including gynecologists, midwives, GPs, practice nurses, physiotherapists, pharmacists, dietetics, specialists, preventive child health care and social workers – share accountability and collaborate towards comprehensive integral preconception care. Important prerequisites for this approach are third party reimbursement, a local cooperation network and an adequate referral system. Ultimately, PCC should be incorporated in every suitable health encounter by routinely and opportunistically discussing childbearing plans and preconception health. To accomplish this goal, professionals need to be provided with proper knowledge, skills and tools to facilitate the uptake of PCC, while the awareness of prospective parents needs to be enhanced by promotional campaigns and activities.

### Findings in relation to the literature

Lack of finances and time was confirmed to be an important barrier for the delivery of PCC in several other studies [13, 15–18, 30, 32]. Morgan et al. reported that half of obstetricians/gynecologists agree that they do not have enough time to provide PCC to women of childbearing age and that time devoted to PCC is not reimbursed [16]. In a study by Mazza et al. GPs estimated that following PCC guidelines would take longer than standard consultations and has the potential to burden primary care clinics [15]. In accordance with our results, van Voorst et al. indicated that adequate financing is a prerequisite for the delivery of PCC [18]. Without third party reimbursement providers have limited means to provide PCC. Next, our study points out that unclear allocation of responsibilities regarding the referral and execution of PCC consults is a second constraint for the delivery of PCC. Previous research confirms that one of the major challenges is how to target prospective parents as they often do not disclose their pregnancy plans to healthcare providers [8]. Since almost all women of reproductive age presenting to primary care settings are candidates for PCC, health care providers such as GPs are essential and critical for referral for PCC [30, 33]. Despite the broad support from different primary care professionals for this meeting on the local implementation of PCC, there was a lack of involvement of GPs, which corresponds with the finding of Van Voorst et al. that currently only 40% of GPs in the Netherlands think they should be responsible for PCC [18]. Heyes et al. performed a study in the UK and found similar results, as GPs did not prioritize PCC [13]. As mentioned earlier, differences in healthcare systems hinder measuring and comparing the provision of PCC between regions [6, 7, 34]. Therefore, the most pragmatic approach is to appoint the most engaged healthcare profession within the regional/national setting, for example midwives within the context of the Netherlands, to be responsible for the provision of individual preconception care consultations. Meanwhile the other healthcare disciplines should share accountability and responsibility in the role of referrer within a clear local cooperation network. It has previously been advocated to address “every woman, every time”, as there is an opportunity to address reproductive life plans and preconception health during every health encounter by—not exclusively the GP but rather—a range of health care professionals [3, 34–36]. Along this line, we recommend to incorporate PCC by routinely discussing preconception health during suitable health encounters with both male and female clients of reproductive age, by healthcare professionals from a wide range of disciplines—from ob/gyns, midwives, GPs and practice nurses to physiotherapists, pharmacists, dietetics, specialists, preventive child health care and social workers. Previous research has shown that although the majority of professionals have positive attitudes towards PCC, there is a lack of knowledge and need for education and postgraduate courses [13, 16, 17, 32]. Professionals need to be provided with proper knowledge, skills and tools to accomplish comprehensive integral preconception care in which childbearing plans and preconception health are opportunistically addressed, to every couple, every time.

### Strengths and limitations

A strength of this study is the collaboration of representatives of all relevant occupational groups, as well as stakeholders towards an integrated PCC approach. To attain multidisciplinary consensus, the five discussion groups were diversely composited with an equal distribution of participants from different professionalisms among the groups. Secondly, the use of the Nominal Group Technique (NGT) asserts the engagement of participants in data analysis and decision making, which enlarged the validity of our study findings [29]. The NGT limits possible researcher bias, as data interpretation is for the larger part performed by participants. Obtaining data saturation was another strength of this study, as participants addressed all pre-defined themes during the discussion sessions, while no new themes emerged.

A limitation of this study was the participation of only two GP’s in the discussion groups, which illustrates the limited engagement of GP’s with preconception care. Another limitation is the use of five different moderators to guide the discussion groups, as these took place simultaneously. To minimize inter-group variety, a detailed protocol was used (Table 2) and a preparation session was held. More importantly, the use of a local setting could potentially limit the generalizability of our study results. Although the population of Zeist is for the most part representative for the Netherlands, educational and income levels are proportionally high. The purpose of this approach was to obtain regional agreement and obtain solutions that are locally applicable, as they are tailored to the local context and prevailing bottlenecks. Yet, the specific local context and interaction between health care providers will have influenced the results. However, we think that replicating this process in other regions will result in largely similar barriers and solutions, as no new topics emerged and most barriers also apply to other regions. The ranking and severity of perceived bottlenecks is expected to vary between regions, due to local circumstances.

## Conclusion

In this study, a multidisciplinary providers’ perspective on the delivery of preconception care is provided. The current provision of PCC is challenging, due to lack of awareness, the absence of a costing structure and unclear allocation of responsibilities. The findings of this study call for a tailored approach in which interdisciplinary arrangements for collaboration and referral are set up within the local primary care setting. For successful implementation of PCC, it is recommended to 1) settle a costing structure by means of third party reimbursement, 2) improve collaboration by means of a local cooperation network and adequate referral system, 3) invest in education, tools and logistics and 4) increase uptake rates by the routine opportunistic offer of PCC and promotional campaigns.
